# Comprehensive analysis of gut microbiota and fecal metabolites in patients with autism spectrum disorder

**DOI:** 10.3389/fmicb.2025.1557174

**Published:** 2025-04-25

**Authors:** Ruijuan Zheng, Silu Huang, Pengya Feng, Simeng Liu, Miaomiao Jiang, Huijuan Li, Pengyuan Zheng, Yang Mi, Enyao Li

**Affiliations:** 1 Department of Child Rehabilitation, The Fifth Affiliated Hospital of Zhengzhou University, Zhengzhou, China; 2 Henan Key Laboratory of Helicobacter Pylori and Microbiota and Gastrointestinal Cancer, Marshall B. J. Medical Research Center of Zhengzhou University, The Fifth Affiliated Hospital of Zhengzhou University, Zhengzhou, China; 3 Department of Gastroenterology, The Fifth Affiliated Hospital of Zhengzhou University, Zhengzhou, China

**Keywords:** autism spectrum disorder, gut microbiota, 16S rRNA sequencing, metabolomics, biomarker

## Abstract

**Introduction:**

Autism spectrum disorder (ASD) is a neurodevelopmental disorder characterized by deficits in social communication and the presence of restricted, repetitive behaviors or interests. Studies have revealed that gut microbiota and their metabolism play important roles in ASD, and become the underlying mechanisms of ASD.

**Methods:**

In this study, we performed long-read 16S rRNA sequencing and untargeted metabolomics to comprehensively characterize the profiles of gut microbiota and fecal metabolites in 34 ASD patients and 18 healthy controls. The associations between gut microbiota, fecal metabolites and clinical symptoms were analyzed to screen related biomarkers for ASD.

**Results:**

The results showed the similarity of the overall microbial richness and diversity between ASD patients and controls, however, some specific bacterial taxa exhibited significant differences, including *Klebsiella* and *Escherichia-Shigella* at genera level, and *Clostridium-sporogenes*, *Escherichia-coli-O157H7* and *Bacteroides-ovatus* at species level. The fecal metabolomics validated that a lot of metabolites had significantly differential levels, including a series of organic acids, amino acids and dopamine.

**Discussion:**

The associations of gut microbiota and fecal metabolites might shed new light on the pathogenesis of ASD and help us to understand the importance of gut microbiota as potential biomarkers and therapeutic targets in the development of ASD.

## Introduction

Autism spectrum disorder (ASD) is a group of neurodevelopmental disorders characterized by impairment in social communication, narrow range of interests or activities, and repetitive stereotyped behavior (Valentino et al., 2021). Patients typically exhibit symptom onset between 6 and 24 months, with some individuals initially displaying normal development followed by degenerative changes occurring at 24–36 months, characterized by a decline in language and social skills. The prevalence rate of ASD has been increasing in recent years (Okada and Ozaki, 2015; Shaw et al., 2021), however, the specific pathogenesis of ASD is still unclear.

The presence of gastrointestinal symptoms, including constipation, abdominal pain, and diarrhea, is frequently observed in individuals with ASD (Bauman, 2010; Bresnahan et al., 2015; Cermak et al., 2010; Leader et al., 2022). Therefore, it can be inferred that gut microbiota plays a crucial role in maintaining human health. In fact, the genome of gut microbiota also influences the expression of human proteins and metabolic genes, and any disruption to the homeostasis of gut microbiota could have detrimental effects on human health (Srivastava et al., 2021). For instance, an imbalance in the microbiota is one of the contributing factors to Alzheimer’s disease, leading to increased blood-brain barrier permeability and intestinal permeability (Jiang et al., 2017). Su et al. (2024) reported that the potential application of multi-kingdom and functional gut microbiota markers as non-invasive diagnostic tools in ASD. In the comprehensive analysis of ASD pathogenesis, studies also have revealed notable disparities in the composition of gut microbiota when compared to typically developing individuals (Sharon et al., 2019). Lou et al. (2022) also demonstrated that a signature of the combination of Veillonella and Enterobacteriaceae, and 17 microbial metabolic functions efficiently discriminated ASD from neurotypical subjects. In a study conducted on patients with ASD, gut microbiota dysbiosis was observed to impact the normal functioning of intestinal epithelial cells, toxin metabolism, food metabolism and nutrient absorption, as well as the immune system (Tonacci et al., 2019). These findings suggested important roles of human gut microbiota in ASD patients, therefore, the investigation about the gut microbiota might provide valuable insights for revealing the underlying pathogenesis of ASD.

The gut microbiota establishes a metabolic axis, connecting the gut, liver, brain, and other organs through the host-microbiota co-metabolic structure (Yadav et al., 2022). This intricate network actively participates in regulating the host’s systemic metabolism. Previous research has demonstrated that ASD children with gastrointestinal symptoms exhibited distinct differences in their gut microbiota composition compared to healthy individuals, and showed reduced levels of short-chain fatty acids (SCFAs) (Zhao et al., 2023). Furthermore, some studies have suggested that SCFAs might have a beneficial or detrimental effect on the gut and neurological development of ASD patients (Lagod and Naser, 2023; Taniya et al., 2022). Abnormal connectivity of neural circuits can arise from an imbalance in brain serotonin (5-HT) levels (Chandana et al., 2005). The synthesis and secretion of 5-HT could be regulated by gut microbiota, and native *Bacillus* promoted gut chromaffin cells to synthesize 5-HT (Gershon, 1999), in addition, *Streptococcus* and *Enterobacter* could also synthesize 5-HT (Bai et al., 2022; Hata et al., 2017; Yano et al., 2015). Therefore, the metabolism of gut microbiota plays a pivotal role in human health, and investigating the interaction of microbiota and fecal metabolism holds promise for unraveling the mechanisms of ASD.

In this study, we have observed significant alterations in the gut microbiota and metabolites of between 34 ASD patients and 18 healthy controls (HC) by 16S ribosomal RNA (rRNA) sequencing and fecal metabolomics. Specifically, the microbial richness and diversity was similar between ASD and HC, accompanied by differences in some specific bacterial taxa, including *Klebsiella* and *Escherichia-Shigella* at genera level. Importantly, metabolomics revealed some metabolites were significantly different, including multiple organic acids, amino acids and dopamine. We also investigated the association between gut microbiota and fecal metabolites to gain further insights into the pathogenesis of ASD. This study highlighted potential roles of gut microbiota and fecal metabolites for differentiating ASD and HC as non-invasive diagnostic biomarkers, and provided important information for revealing the pathogenesis of ASD.

## Materials and methods

### ASD patients and sample collection

We recruited 34 autistic subjects between 2020 and 2022, at the time of the Autism Diagnostic Observation Schedule (ADOS, 2nd Edition) test and language of at least one word were included from the Fifth Affiliated Hospital of Zhengzhou University. Eighteen age- and sex-matched healthy volunteers were accepted as neurotypical controls (NT). The diagnosis of autism was established according to DSM-V (Diagnostic and Statistical Manual of Mental Disorders, 5th Edition) (First, 2013) and ICD-10 (International Statistics Classification of Diseases and Related Health Problems, 10th Revision) (Bramer, 1988) by two experienced child neuropsychiatrists. Neurotypical controls were typically developing children, without an autism diagnosis and not directly related to an autistic individual. The exclusion criteria included a history of nutritional supplements and special diets, presence of significant physical abnormalities, and neurological disorders of known etiology. Participants in this study were not treated with antibiotics, antifungals, probiotics, or prebiotics for at least 3 months before sampling. All methods were carried out in accordance with the relevant guidelines and regulations. All experimental protocols were approved by the Ethical Committee of the Fifth Affiliated Hospital of Zhengzhou University (No.2016-1001). Informed written consent was obtained from the parents and/or legal guardians of the enrolled participants. Fresh fecal samples were collected from 34 ASD patients and 18 HC at the Fifth Affiliated Hospital of Zhengzhou University. The fecal samples were stored at −80°C until following use.

### IgG food intolerance testing

Food-specific IgG antibodies were detected using Enzyme-linked immunosorbent test that measures IgG levels against 14 food substances using a single blood sample. This testing procedure involved incubation of the patient serum sample for 30 min at room temperature on a glass microscope slide containing a microarray of 14 food extracts. After this primary incubation, the slide was washed to remove unbound proteins. Following this anti-human IgG conjugated to horseradish peroxidase was added to the slide and incubated for another 30 min at room temperature. The slide was washed again to remove the unbound conjugate. After washing 3, 3’, 5’, 5’-tetramethybenzidine substrate was added to detect specific antibody binding. A third incubation was performed for 10 min and then the slide was washed with distilled water. Finally, the slides were centrifuged and scanned by a high-resolution flatbed scanner associated with computer software that interprets the optical densities of the samples. IgG levels less than 50 U/mL were considered negative, whereas levels either equal to or more than 50 U/mL were considered positive. Values of food specific IgG antibodies either equal to or more than 50 U/mL were included in the study.

### Event-related potentials recordings

ERP recordings were made using a 2-channel Electrical Geodesics Incorporated (EGI) system, Nippon Optronics. Only data from the relevant electrodes (where maximum amplitude was reached) were analyzed for the present study, i.e., midline electrode FCz for mismatch negative (MMN) and P3A amplitude, and midline electrode Pz for P3B amplitude, with average reference. A standard method of data processing was used: Neuro Workbench software was used for processing the EEG signals. To allow easier file handling, the data was first down-sampled from the original 500 to 250 Hz. Second, data were corrected for eye-artifacts using the adaptive method of BESA. Third, the data were epoched (from 100 ms pre-stimulus to 900 ms post-stimulus) and corrected for movement and other paradigm unrelated artifacts, by removing those epochs that contained amplitude differences that exceeded 75 μV between the maximum and minimum in the for MMN assessment relevant time window. Last, data were band-pass filtered (0.5–40 Hz). MMN was expressed as a subject’s average ERP to each of the three deviant stimuli from which the average ERP to the standard stimuli was subtracted. MMN was scored as an individual’s maximum negative voltage appearing in the EEG within a time window between 130 and 230 ms for the beep stimulus, and between 140 and 260 ms for the other two deviant types. Similarly, an individual’s P3a amplitude was scored as the maximum positive amplitude in a time window between 200 and 370 ms (all three types of deviants) while the P3B amplitude was assessed as the maximum positive amplitude between 330 and 600 ms.

### Long-read 16S rRNA sequencing

Total bacterial DNA was extracted from each fecal samples using the QIAamp DNA stool minikit (Qiagen, Germany). The 16S rRNA amplicon sequencing was performed on MinION nanopore sequencer (Oxford Nanopore Technologies, UK). The amplicon library was prepared using the 16S Barcoding Kit 1–24 (SQK-16S024, Oxford Nanopore Technologies, UK) according to the instructions. For the PCR amplification and barcoding, 15 ng of template DNA were added to the LongAmp Hot Start Taq 2X Master Mix (New England Biolabs, UK) as the manufacturer’s instructions. Then, the barcoded amplicons were purified using the AMPure XP beads (Beckman Coulter, USA) as the Nanopore’s instructions. Samples were then quantified by Qubit fluorometer (Life Technologies, USA) and pooled in an equimolar ratio to a total of 50–100 ng in 10 μL. The pooled library was then loaded into an R9.4.1 flow cell and run according to the Nanopore’s instructions. MINKNOW software 19.12.5 was used for data acquisition.

### 16 S rRNA sequencing data analysis

Raw fastq sequences were base called by using Guppy. Sequencing adapters were then removed from sequences with Porechop v0.2.4. The resulting reads were classified taxonomically with Kraken v1.1.1 (Wood et al., 2019). The Pavian web application^1^ was utilized to transform Kraken outputs into spreadsheets of taxa counts and relative abundances per sample at phylum, family, genus, and species levels as described previously (Soriano et al., 2022).

Alpha diversity, including the observed species, Chao1, ACE, Shannon, Simpson, and coverage indices, were calculated using Mothur software. The beta diversity analysis was analyzed for the differences of species diversity in different samples, consisted of principal component analysis (PCA), principal-coordinate analysis (PCoA), and nonmetric multidimensional scaling (NMDS). Linear Discriminant Analysis Effect Size (LEfSe) analysis was employed to identify species marker with differential abundances among these two groups.

### Fecal metabolomics

The fecal samples stored at −80°C were thawed on ice. A 400 μL solution (methanol: water = 7:3) containing internal standard was added into 20 mg sample, and then vortexed for 3 min. The samples were sonicated in ice bath for 10 min and vortexed for 1 min, and then placed in −20°C for 30 min. The samples were further centrifuged at 12,000 rpm for 10 min at 4°C. And the sediment was removed, then the supernatant was centrifuged at 12,000 rpm for 3 min at 4°C. A 200 μL aliquot of supernatant was transferred for following liquid chromatography-mass spectrometry (LC-MS) analysis.

The fecal metabolites were quantitated in an ultra-performance liquid chromatography (UPLC) system (ExionLC, SCIEX, United States) coupled to a tandem mass spectrometry system (TripleTOF 6600, SCIEX, United States). The analytical conditions were as follows, UPLC: column, Waters ACQUITY UPLC HSS T3 C18 (1.8 μm, 2.1 mm × 100 mm); column temperature, 40°C; flow rate, 0.4 mL/min; injection volume, 2 μL or 5 μL; solvent system, water (0.1% formic acid): acetonitrile (0.1% formic acid); gradient program, 95:5 V/V at 0 min, 10:90 V/V at 10.0 min, 10:90 V/V at 11.0 min, 95:5 V/V at 11.1 min, 95:5 V/V at 14.0 min.

The Triple TOF mass spectrometer was used for its ability to acquire MS/MS spectra on an information-dependent basis during an LC/MS experiment. In this mode, the acquisition software (TripleTOF 6600, SCIEX, United States) continuously evaluates the full scan survey MS data as it collects and triggers the acquisition of MS/MS spectra depending on preselected criteria. The electrospray ionization (ESI) source conditions were set as following: Ion source gas 1 as 50 Psi, Ion source gas 2 as 50 Psi, Curtain gas as 25 Psi, source temperature 500°C, Ion Spray Voltage Floating (ISVF) 5,500 V or −4,500 V in positive or negative modes, respectively.

### Metabolomics data analysis

MS raw data files were converted to the ABF format using AbfConverter.4.0.0 and processed using MS-DIAL, generating a data matrix. Structural identification of metabolites was performed based on comparison of the molecular ion mass and MS/MS segments with Human Metabolome Database.^2^

The population distribution of all fecal samples was evaluated by unsupervised PCA using scaled data. The differential metabolites between these two groups were analyzed by orthogonal partial least-squares discriminant analysis (OPLS-DA). Identification of differential metabolites was based on variable importance for the projection (VIP) score (VIP > 1), absolute Log2FC (|Log2FC| > 1.0) and p value < 0.05. The identified metabolites were annotated using KEGG Compound database,^3^ and annotated metabolites were then mapped to KEGG Pathway database^4^ for further pathway enrichment analysis.

### Statistical analysis

All statistical analysis was conducted with R-4.3.1.^5^ Wilcoxon rank sum test was used for the comparison of alpha diversity index between the two groups. The Kruskal-Wallis (KW) rank sum test was performed to identify abundance differences in specific bacterial taxa in LEfSe analysis. The Spearman correlation coefficient was analyzed between the gut microbiota and fecal metabolites. A *p*-value < 0.05 was considered to be statistically significant.

## Results

### Alpha diversity of gut microbiota in ASD and healthy controls

**FIGURE 1:**
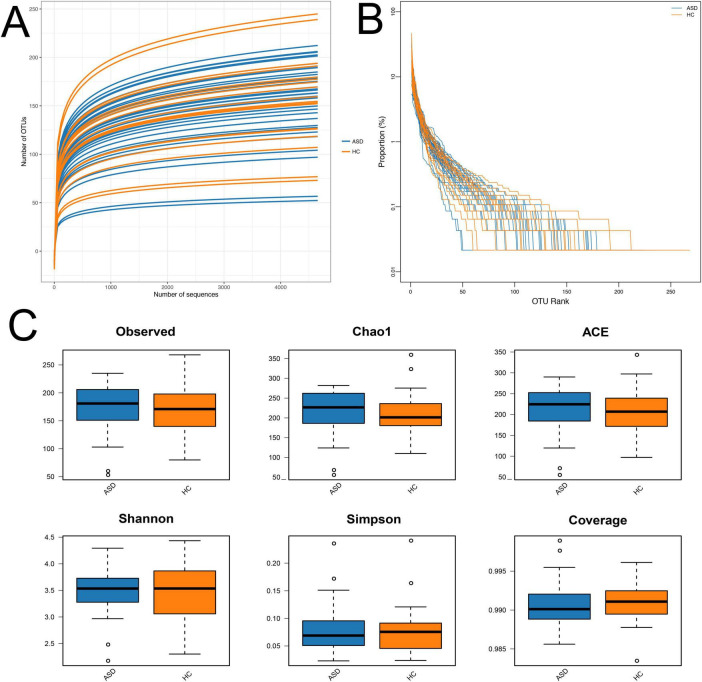
Alpha diversity analysis. **(A)** Rarefaction curves; **(B)** rank-abundance distribution curve; **(C)** diversity and richness of gut microbiota in ASD and HC, including observed species, Chao1, ACE, Shannon, Simpson, and Coverage. There were no significant differences in the microbial richness and diversity between ASD and HC. ASD, autism spectrum disorder; HC, healthy control.

In order to assess the changes in microbial community structure between 34 ASD patients and 18 healthy controls (HC), fecal microbial DNA was extracted for next-generation 16S rRNA sequencing by using the Nanopore MinION platform. The Nanopore sequencing reads the entire length of DNA fragment included in the libraries from short to ultralong (longest > 4 Mb), therefore, it has shown a more accurate taxonomic classification at the species level than the regular 16S rRNA sequencing by Illumina MiSeq or Hiseq platform (Nygaard et al., 2020). We firstly performed the microbial alpha diversity analysis based on the sequencing data. The rarefaction curve demonstrated that all samples achieved high coverage, indicating sufficient sequencing depth covered all species within the samples (Figure 1A). Moreover, the flatness of each group’s rarefaction curve suggested that additional sampling would yield only a marginal number of new operational taxonomic units (OTUs), implying an appropriate number of extracted samples (Figure 1A). The curve of most ASD samples exhibited a relatively low slope compared with that of HC, suggested a significantly lower quantity of OTUs in ASD than HC (Figure 1A). The rank-abundance curve served as a representation of species abundance and evenness in the samples. Compared to HC, the decline in the curve for ASD was more pronounced, and also exhibited a narrower range on the horizontal axis. These findings suggested lower species richness in ASD (Figure 1B).

The analysis indexes of alpha diversity commonly contain observed species, Chao1, abundance-based coverage estimator (ACE), Shannon, Simpson, and Coverage. The microbial richness was evaluated by analyzing the observed OTUs, Chao1, and ACE indices, while microbial diversity was evaluated by Shannon, Simpson, and Coverage indices. The results showed comparable microbial richness and diversity between ASD group and HC group, which did not have significant differences (Figure 1C).

### Beta diversity of gut microbiota in ASD and healthy controls

**FIGURE 2:**
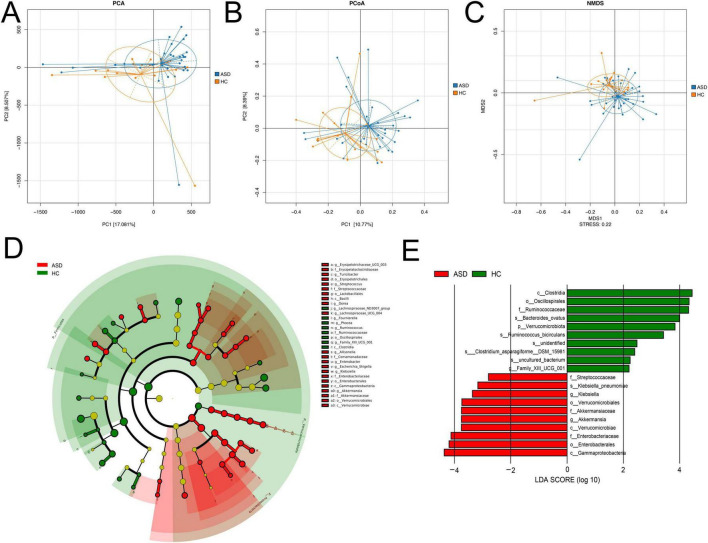
Beta diversity analysis. **(A)** PCA, **(B)** PCoA, **(C)** NMDS analysis results based on the OTU matrix of ASD patients and healthy controls. **(D)** The cladogram from LEfSe analysis revealed differential microbial taxonomy in ASD and HC group. **(E)** Histograms of LDA scores in ASD and HC, with a cutoff value of LDA score (log10) above 2.0.

Beta diversity analysis consisted of principal component analysis (PCA), principal-coordinate analysis (PCoA), and nonmetric multidimensional scaling (NMDS), the latter two were based on Bray-Curtis (Figures 2A–C). These results revealed the similarities and differences between these two groups based on the overall OTUs (Figures 2A–C).

In order to identify the specific bacterial taxa between ASD and control, the microbial community composition was compared by using LEfSe analysis (Figure 2D), which provided taxonomic information and abundance differences among species in each group. The cladogram represented phylum, class, order, family, genus, and species from the center to the outside in taxonomy, respectively. At each classification level, the size of a node representing a species was positively correlated with the abundance of the species, while yellow nodes indicated the species with no significant differences (Figure 2D). The cladogram analysis revealed 30 taxonomies of gut bacterial species had significant variations between ASD and HC, with 22 taxonomies in ASD and eight in HC. For ASD, five key families were identified, including Erysipelatoclostridiaceae, Streptococcaceae, Comamonadaceae, Enterobacteriaceae, and Akkermansiaceae (Figure 2D). Additionally, the main taxonomies with statistically significant differences between these two groups were determined by using LDA (LDA score > 2, *p* < 0.05). In ASD, the top biomarkers contained *Klebsiella*, *Akkermansia*, and Streptococcaceae (Figure 2E).

### Changes of microbial community at the OTU level

**FIGURE 3:**
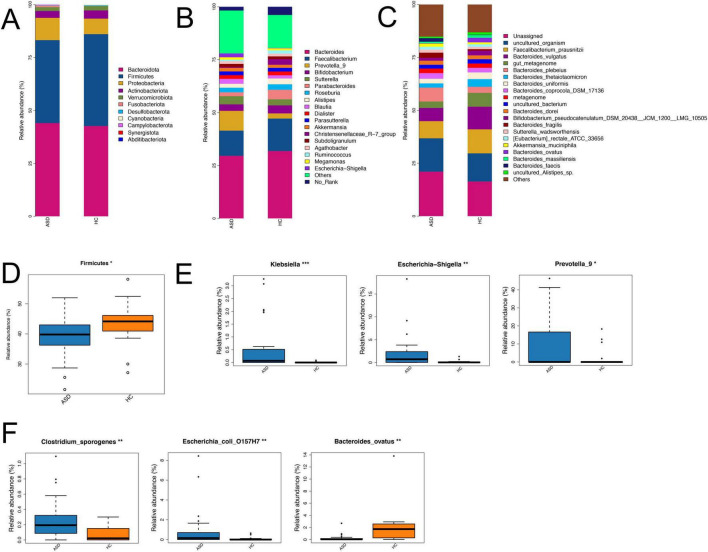
Differences in the composition of gut microbiota between ASD and HC across various taxonomic levels. Bar plots showed the composition differences of gut microbiota at the phylum **(A)**, genus **(B)** and species **(C)** levels in two groups. Boxplots revealed the relative abundance of several differential gut microbiota at the phylum **(D)**, genus **(E)** and species **(F)** levels between ASD and HC. *p < 0.05, **p < 0.01; ***p < 0.001.

To better reveal the changes of the gut microbial community in ASD compared with HC, further analysis showed that Bacteroidota, Firmicutes, and Proteobacteria dominated the gut microbiota at the phylum level (Figure 3A), only Firmicutes had significantly lower relative abundance in ASD (39.17% in ASD and 43.43% in HC, Figure 3D). At the class level, Bacteroidaceae, Clostridia, and Gammaproteobacteria were the major classes in both groups (Supplementary Figure S1A). Among them, Gammaproteobacteria, Clostridia and Bacilli had significant differences between ASD and HC (Supplementary Figure S1D). At the order level, Bacteroidales, Oscillospirales, and Lachnospirales had higher abundance in two groups (Supplementary Figure S1B), Enterobacterales and Erysipelotrichales were enriched in ASD, while Oscillospirales and Christensenellales were higher in HC (Supplementary Figure S1E). At the family level, Bacteroidaceae, Ruminococcaceae and Lachnospiraceae were major families (Supplementary Figure S1C), Enterobacteriaceae and Prevotellaceae had higher abundance in ASD, Christensenellaceae and Ruminococcaceae were higher in HC (Supplementary Figure S1F). In addition, *Bacteroides*, *Faecalibacterium* and *Prevotella*_9 were the major genera in two groups (Figure 3B), multiple genera showed obvious changes in ASD compared with HC, including *Klebsiella*, *Escherichia-Shigella* and *Prevotella_9* (Figure 3E). Importantly, we also found some species had relative differences between ASD and HC, including *Clostridium-sporogenes*, *Escherichia-coli-O157H7* and *Bacteroides-ovatus* (Figures 3C,F).

### Fecal metabolic profiles in ASD patients and healthy controls

**FIGURE 4:**
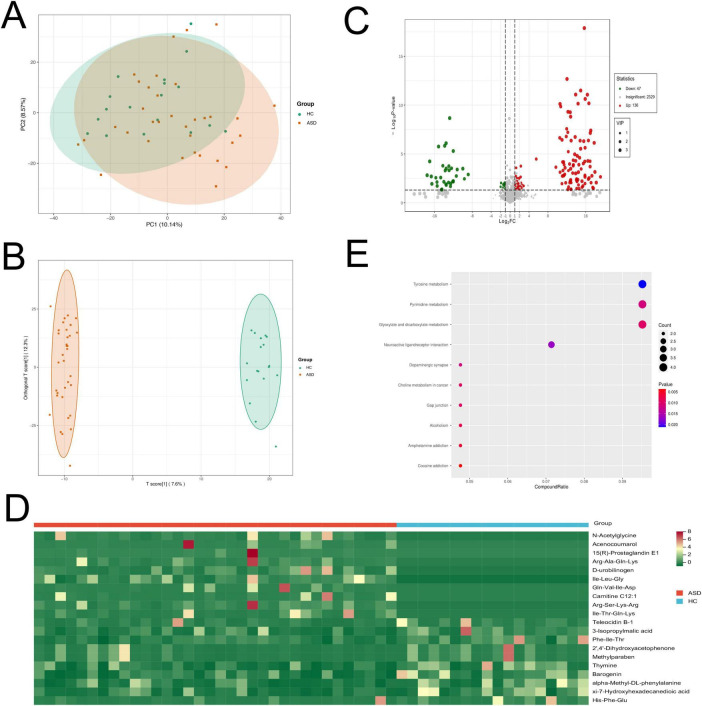
Fecal metabolomics profiles of ASD patients and HC by metabolomics analysis. **(A)** PCA plot of fecal metabolites in ASD and HC. **(B)** Score plot of OPLS-DA revealed that the composition of fecal metabolites were significantly different in ASD and HC. **(C)** Volcano plot showed the number of significantly differential fecal metabolites in ASD compared with HC. **(D)** Heatmap showed the top 10 up-regulated and 10 down-regulated metabolites in ASD compared to HC. **(E)** Pathway analysis showed that differential fecal metabolites were enriched in the related pathways.

To further investigate the fecal metabolic changes between ASD patients and HC, we performed untargeted metabolome profiling in collected feces samples by UPLC-QTOF-MS/MS. The PCA result revealed some differences in the overall fecal metabolite profiles between these two groups (Figure 4A). The supervised OPLS-DA method demonstrated significant alterations in the composition of fecal metabolites in ASD patients (Figure 4B).

The analysis of the OPLS-DA model revealed 183 annotated differential metabolites between ASD and HC, with VIP > 1, and *p* < 0.05. Among these metabolites, there were 136 up-regulated and 47 down-regulated metabolites (absolute |log_2_FC| > 1, Figure 4C). The differential metabolites mainly contained multiple organic acids, amino acids, hormones and their related metabolites or derivatives (Supplementary Figures S2A,B). The top up-regulated metabolites were N-acetylglycine, acenocoumarol, 15(R)-prostaglandin E1, Arg-Ala-Gln-Lys and D-urobilinogen in ASD patients (Figure 4D). Interestingly, we also found that dopamine was significantly up-regulated in ASD patients compared to HC.

The metabolic pathway enrichment analysis was performed using the KEGG database. The results revealed differential metabolites were significantly enriched in some neural pathways, including neuroactive ligand-receptor interaction and dopaminergic synapse. Other related pathways contained tyrosine metabolism, pyrimidine metabolism and glyoxylate and dicarboxylate metabolism (Figure 4E). These findings suggested a notable transformation in fecal metabolites among individuals with ASD compared to HC, highlighting the potential of metabolic profiling as an effective tool for distinguishing between these two groups.

### The correlation analysis between fecal metabolomics changes and gut microbiota composition in ASD patients and healthy controls

**FIGURE 5:**
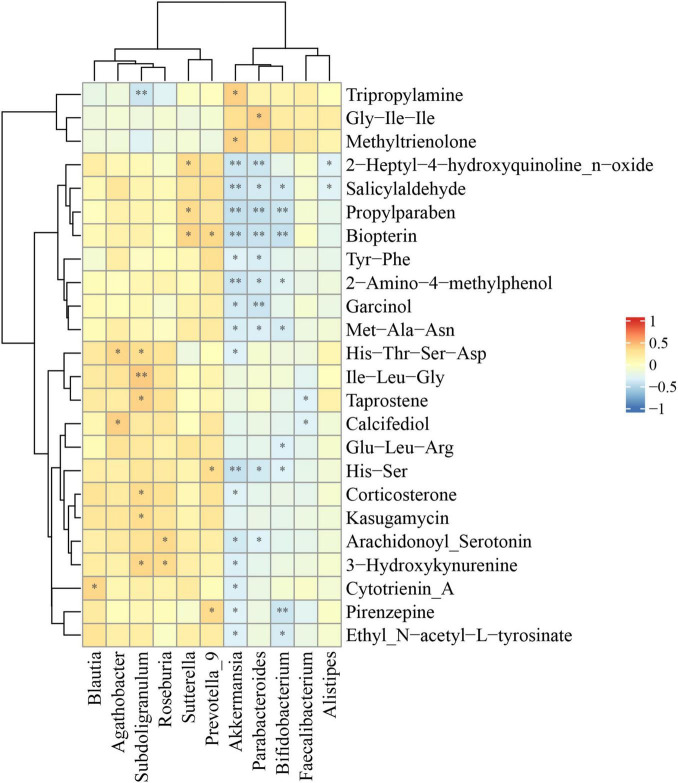
Correlations between relative abundance of gut microbiota and differential fecal metabolites. The Spearman correlation coefficient was analyzed between the gut microbiota at the genus level and differential fecal metabolites in ASD compared to HC. The data was shown via heatmap, red and blue represented positive and negative correlations with different correlated degrees, respectively. *p < 0.05, **p < 0.01.

In order to explore the possible sources of the fecal metabolites, we analyzed the correlations between fecal metabolites and gut microbiota at the genus level in the comparison of ASD and HC (Figure 5). We found that there was a strong positive correlation between *Subdoligranulum*, *Sutterella*, *Prevotella_9* and some significantly differential metabolites, including Ile−Leu−Gly, biopterin and 3−hydroxykynurenine. In addition, *Akkermansia*, *Parabacteroides*, *Bifidobacterium* and multiple differential metabolites had significantly negative correlation, including salicylaldehyde, propylparaben, biopterin, 2−amino−4−methylphenol, Met−Ala−Asn, and His−Ser (Figure 5).

### The correlation analysis between clinical symptoms and gut microbiota composition or fecal metabolomics in ASD patients

**FIGURE 6:**
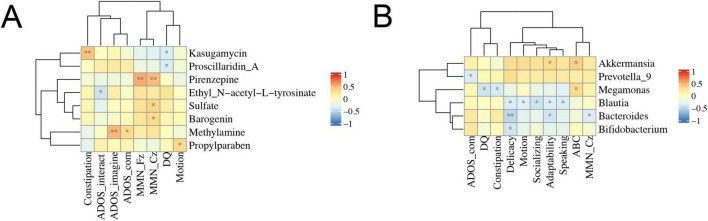
Correlations between clinical symptoms and gut microbiota composition or fecal metabolomics. The Spearman correlation coefficient was analyzed between clinical symptoms and gut microbiota composition **(A)** or fecal metabolomics **(B)** in ASD patients. The data was shown via heatmap, red and blue represented positive and negative correlations with different correlated degrees, respectively. *p < 0.05, **p < 0.01; ADOS_com, ADOS_communication; DQ, development quotient.

Next, we intended to analyze the correlations between the composition of gut microbiota or metabolic products and the severity of clinical symptoms, including ADOS test, ERP recordings, Autism Behavior Checklist (ABC) score and food intolerance in ASD patients (Figure 6). The results showed that there were significant negative correlations between *Blautia* and delicacy, motion, socializing, adaptability, and speaking (Figure 6A). In metabolic products, pirenzepine was significantly correlated with MMN_Fz and MMN_Cz, while methylamine was significantly correlated with ADOS-imagine and ADOS-communication (Figure 6B).

## Discussion

Gut microbiota exerts a significant effect on various human diseases, such as cancer (Chen et al., 2017; Wong and Yu, 2019; Zeng et al., 2017), autoimmune disorders (Aarts et al., 2017; Almonacid et al., 2017), and ASD (Coretti et al., 2018). Notably, emerging research has elucidated the bidirectional communication system known as the “microbiota-gut-brain axis,” which serves as a vital conduit between gut and brain (Cooper, 2018; Mayer et al., 2022; Tache and Saavedra, 2022). More importantly, it has been revealed that gut microbiota possess the ability to modulate metabolite levels, it is advantageous to elucidate the mechanism of gut microbiota affecting ASD through the combined analysis of gut microbiota and their characteristic metabolites. Our investigation revealed discernible disparities in gut microbiota composition between ASD patients and healthy individuals, encompassing distinctive microbiota such as *Klebsiella*, *Escherichia-Shigella*, *Bacteroides-ovatus*, and *Clostridium-sporogenes*. *Klebsiella*, a facultative anaerobe, is among the prevalent pathogenic bacteria in the intestinal microbiota and has developed resistance. It is closely associated with intestinal diseases, as studies have detected *Klebsiella* in the feces of patients with diarrhea, exhibiting a high resistance rate of 97.67% to azithromycin and sulfamethoxazole (Zhang et al., 2018). Furthermore, terizocin induced a rapid proliferation of *Klebsiella* in the intestine, disrupting the stability of the intestinal microbiota (Kienesberger et al., 2022). *Klebsiella* could act as a persistent reservoir bacterium by integrating its genes into other bacterial strains to prevent secondary infections and reduce transmission of highly resistant pathogens between and within patients (Osbelt et al., 2021; Sun et al., 2019). Additionally, research indicated that *Klebsiella* species are linked to primary sclerosing cholangitis (Osbelt et al., 2021). Disruptions in the intestinal microbiota were correlated with an increased proportion of *Escherichia-Shigella* and elevated levels of tumor necrosis factor alpha (TNF-α) and interleukin 6 (IL-6) in blood (Li et al., 2022). The dysbiosis of the intestinal tract was characterized by a significant expansion of *Escherichia-Shigella* species, serving as a marker for IgAN patients and potentially offering a promising diagnostic biomarker and therapeutic target for IgAN (Zhao et al., 2022). *Bacteroides-ovatus*, a Gram-positive *bacterium* capable of producing spores and causing food poisoning, exhibited growth affected by environmental pH and NaCl (Valero et al., 2020), closely linked to diseases such as colitis in the human intestine (Randazzo et al., 2015). Research indicated that the metabolic products of Bacillus cereus are associated with its ATP-producing oxidation-reduction process, thereby influencing the host’s physiological processes (Liu et al., 2022). Furthermore, studies have demonstrated that introducing the *noxA* gene can enhance *Clostridium-sporogenes* oxygen tolerance for potential cancer treatment (Sadr et al., 2024). Considered the next-generation dominant probiotic, *Clostridium-sporogenes* has the ability to express tumor-specific Thomsen-Friedenreich antigens to prevent cancer occurrence and can be genetically modified for treating intestinal diseases (Hamady et al., 2010; Ulsemer et al., 2013). The growth of *Clostridium-sporogenes* was influenced by changes in host conditions and dietary sources within the intestinal environment (Fultz et al., 2021), it aided in breaking down cellulose into usable glucose while preferring unsubstituted pectin utilization (Centanni et al., 2020; Li et al., 2023).

More importantly, it has been revealed that gut microbiota possess the ability to modulate metabolite levels, it is advantageous to elucidate the mechanism of gut microbiota affecting ASD through the combined analysis of gut microbiota and their characteristic metabolites. Research also suggested that *Clostridium-sporogenes* could alter short-chain fatty acid abundance and neurotransmitter levels in the intestine (Horvath et al., 2022). Furthermore, we identified some metabolite markers associated with these divergent microbial taxa in ASD, thereby facilitating a comprehensive understanding of the pathogenesis underlying ASD and its gastrointestinal manifestations. In current study, we found multiple organic acid and its derivatives had differential levels in feces samples of ASD compared to that of HC. Some studies have shown that urine organic acids could be potential biomarkers for ASD in children (Chen et al., 2019), and urinary organic acids could be used as biomarkers for alterations of gut microbiota in children with ASD (Daneberga et al., 2022; Khan et al., 2022). In a urine metabolite analysis of individuals with ASD in South Africa, it was observed that the concentrations of 3-hydroxy-3-methylglutaric acid, 3-methyglutaconic acid, and ethylmalonic acid were elevated in the ASD cohort (Stathopoulos et al., 2020). In addition, a lot of amino acids and its metabolites showed significant differences between ASD and HC. Similarly, several studies have found that fecal amino acids were abnormally metabolized in ASD compared with corresponding controls (Needham et al., 2021; Zhu et al., 2022). Moreover, amino acids played a crucial role in ASD, and several studies have indicated that the amino acid expression profile in individuals with ASD differed from that of neurotypical individuals (Anastasescu et al., 2024; Liu et al., 2019; van Sadelhoff et al., 2019). Recent study has proposed that fecal amino acids could be potential targets for designing personalized diets to prevent or minimize cognitive impairments associated with ASD (Chamtouri et al., 2023). Research has indicated that individuals with ASD exhibited decreased levels of tryptophan, phenylalanine, and tyrosine in their physiological composition (Randazzo et al., 2023). Furthermore, the interplay between amino acids and urinary organic acids might impact the occurrence of electroencephalographic spike abnormalities in the brains of individuals with ASD (Marcotul Li et al., 2022). Interestingly, we also found the level of dopamine was significantly different in ASD and HC. As we all known, the alterations of dopamine signal have been implied in ASD, and they could be associated with the risk of developing a psychotic disorder in ASD patients (Schalbroeck et al., 2021). Dysregulation of dopamine was prevalent in individuals with ASD, and research indicated an association between the dopamine-3 receptor gene (DRD3) and specific repetitive behaviors observed in individuals with ASD (Kosillo and Bateup, 2021; Staal et al., 2012). More importantly, blocking postsynaptic dopamine and serotonin receptors might be beneficial in children with ASD (McCracken et al., 2002).

In conclusion, the investigation into the disparities in gut microbiota between ASD and HC highlights the prominent microbiota and their metabolites, further elucidates the mechanisms underlying the impact of gut microbiota on ASD, and presents potential targets for therapeutic interventions in ASD patients.

## Data Availability

The genomic data of microbiota presented in the study are deposited in the SRA repository, accession number PRJNA1222609 and the metabolomics data are deposited in MetaboLights repository, accession number MTBLS12220.

## Footnotes

1
https://fbreitwieser.shinyapps.io/pavian/

2
http://www.hmdb.ca/

3
http://www.kegg.jp/kegg/compound/

4
http://www.kegg.jp/kegg/pathway.html

5
www.r-project.org
