# Correction: Comprehensive analysis of gut microbiota and fecal metabolites in patients with autism spectrum disorder

**DOI:** 10.3389/fmicb.2026.1812620

**Published:** 2026-04-07

**Authors:** Ruijuan Zheng, Silu Huang, Pengya Feng, Simeng Liu, Miaomiao Jiang, Huijuan Li, Pengyuan Zheng, Yang Mi, Enyao Li

**Affiliations:** 1 Department of Child Rehabilitation, The Fifth Affiliated Hospital of Zhengzhou University, Zhengzhou, China; 2 Henan Key Laboratory of Helicobacter Pylori and Microbiota and Gastrointestinal Cancer, Marshall B. J. Medical Research Center of Zhengzhou University, The Fifth Affiliated Hospital of Zhengzhou University, Zhengzhou, China; 3 Department of Gastroenterology, The Fifth Affiliated Hospital of Zhengzhou University, Zhengzhou, China

**Keywords:** autism spectrum disorder, gut microbiota, 16S rRNA sequencing, metabolomics, biomarker

In the published article, the sequencing platform Nanopore MinION was mistakenly reported instead of PacBio Sequel II, resulting in several corrections to the **Materials and methods** Section and one correction to the **Results** Section.

A correction has been made to **Materials and methods**, *Long-read 16S rRNA sequencing*, paragraph one:

“For the PacBio long-read sequencing, a full-length 16S rRNA gene was amplified using the universal primers 27F (AGRGTTYGATYMTGGCTCAG) and 1492R (RGYTACCTTGTTACGACTT). Both the forward and reverse 16S primers were tailed with sample specific PacBio barcode sequences to allow for multiplexed sequencing. After that, the amplicons were sequenced on the PacBio Sequel 2 platform (Pacific Biosciences, USA) according to the manufacturer's instructions by Wuhan Frasergen Bioinformatics Co. Ltd. (China).”

A correction has been made to **Materials and Methods**, *16S rRNA sequencing data analysis*, paragraph one:

“The PacBio amplicon data was further processed using the open-source bioinformatics platform, QIIME2 (version 2023.7.0) (Bolyen et al., 2019). The DADA2 R Statistics package (v1.20.0) was used to denoise the reads and create amplicon sequence variants (ASVs) for further taxonomic assignment (Callahan et al., 2016). In addition, SBanalyzer 2.4 software (Shoreline Biome) was used to map circular consensus reads to the Athena database and assign taxonomic identification to all reads (Graf et al., 2021).”

A correction has been made to **Results**, *Alpha diversity of gut microbiota in ASD and healthy controls*, paragraph one:

“In order to assess the changes in microbial community structure between 34 ASD patients and 18 healthy controls (HC), fecal microbial DNA was extracted for next-generation 16S rRNA sequencing by using the PacBio Sequel 2 platform. The PacBio sequencing reads the entire length of DNA fragment included in the libraries from 5 to 10 kb of microbial genome, therefore, it has shown a more accurate taxonomic classification at the species level than the regular 16S rRNA sequencing by Illumina MiSeq or Hiseq platform. We firstly performed the microbial alpha diversity analysis based on the sequencing data. The rarefaction curve demonstrated that all samples achieved high coverage, indicating sufficient sequencing depth covered all species within the samples (Figure 1A). Moreover, the flatness of each group's rarefaction curve suggested that additional sampling would yield only a marginal number of new operational taxonomic units (OTUs), implying an appropriate number of extracted samples (Figure 1A). The curve of most ASD samples exhibited a relatively low slope compared with that of HC, suggested a significantly lower quantity of OTUs in ASD than HC (Figure 1A). The rank-abundance curve served as a representation of species abundance and evenness in the samples. Compared to HC, the decline in the curve for ASD was more pronounced, and also exhibited a narrower range on the horizontal axis. These findings suggested lower species richness in ASD (Figure 1B).”
