# Supplementary figures and images for: Comprehensive analysis of gut microbiota and fecal metabolites in patients with autism spectrum disorder
Source: Front Microbiol. 2025 Apr 25;16:1557174. doi: 10.3389/fmicb.2025.1557174 (PMC12062028; doi:10.3389/fmicb.2025.1557174)

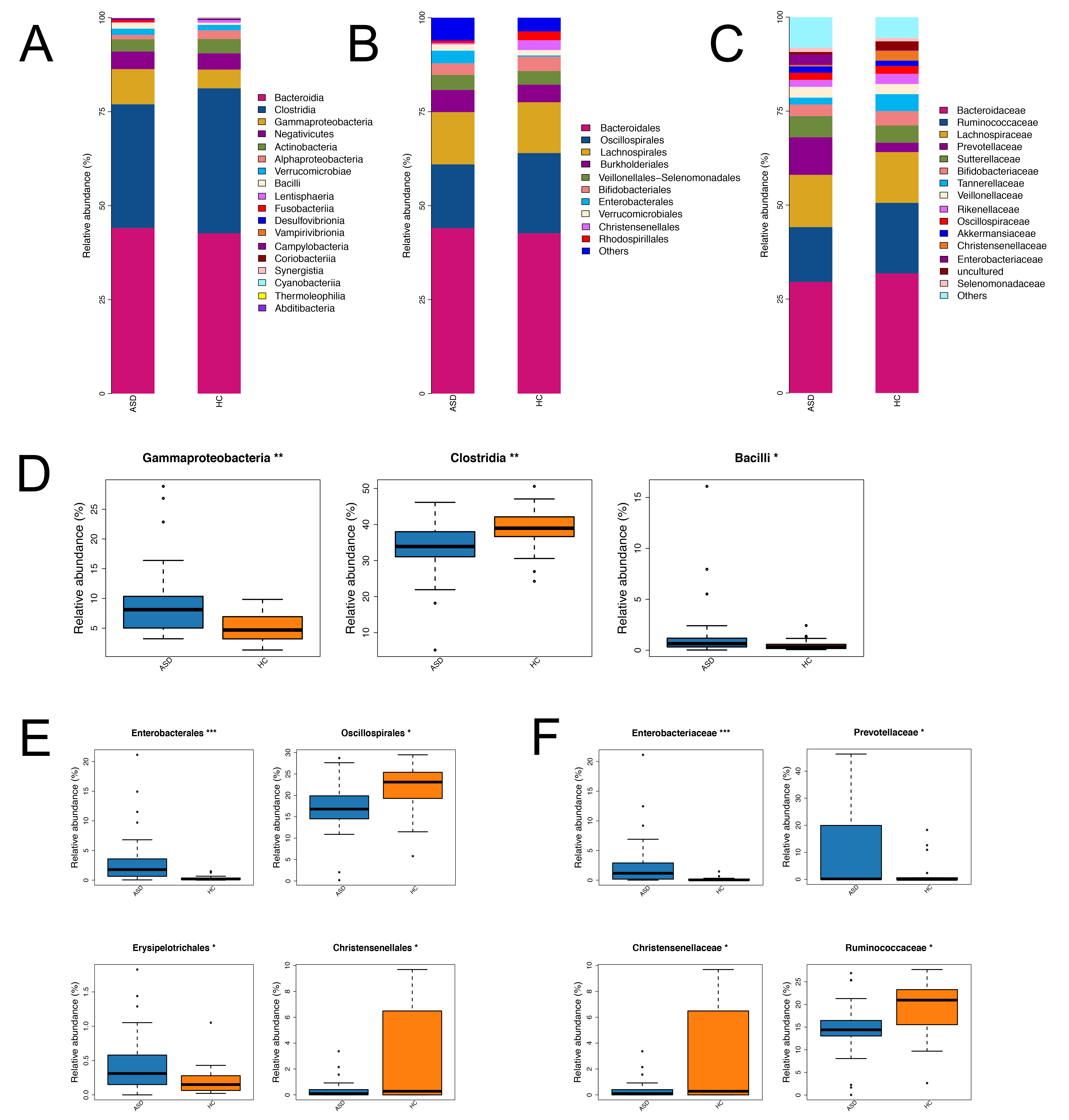

Supplement: Supplementary file 1 [file Image_1.tif]

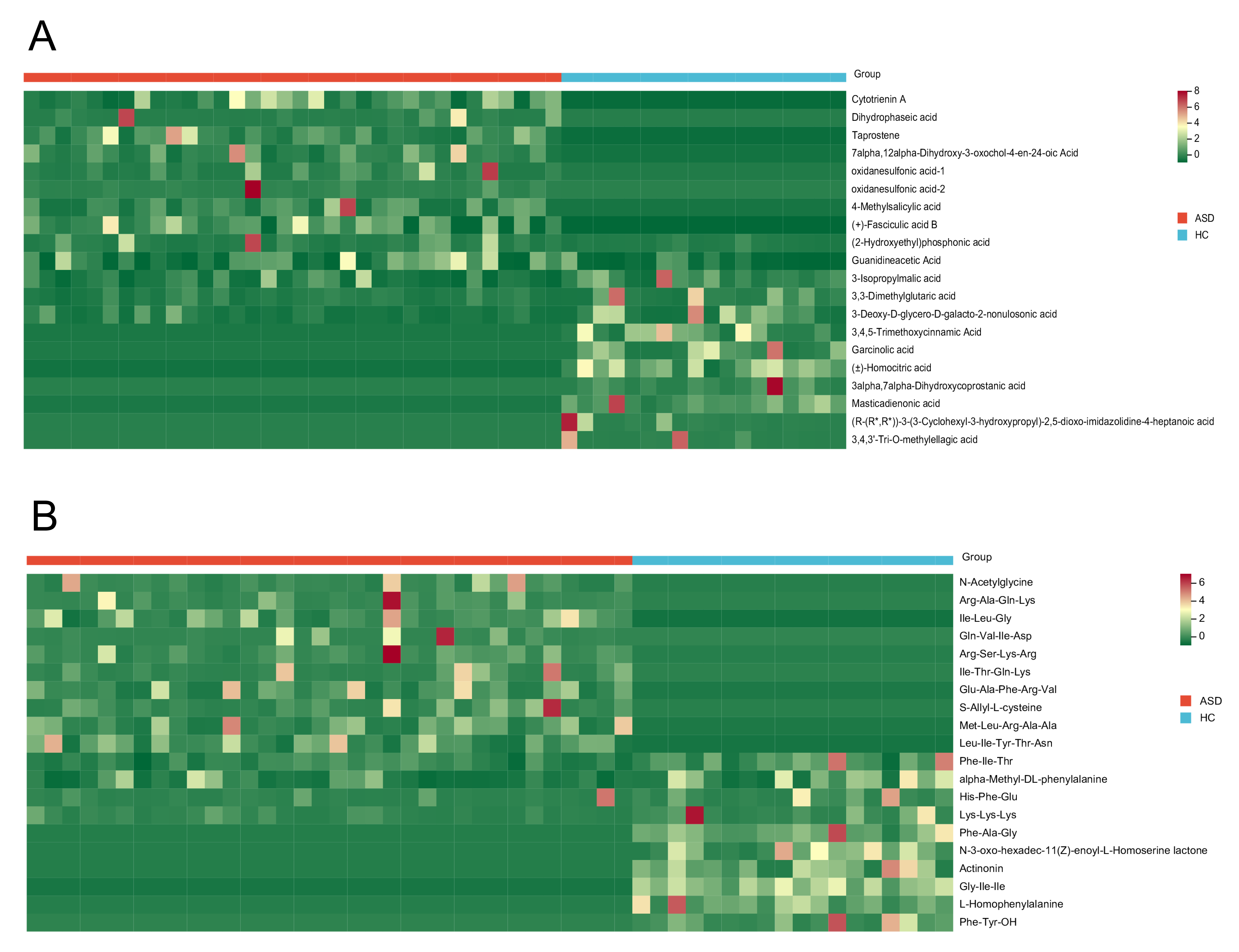

Supplement: Supplementary file 2 [file Image_2.tif]
