# Supplementary material for: Comprehensive analysis of gut microbiota and fecal metabolites in patients with autism spectrum disorder
Source: Front Microbiol. 2025 Apr 25;16:1557174. doi: 10.3389/fmicb.2025.1557174 (PMC12062028; doi:10.3389/fmicb.2025.1557174)
Supplement: Supplementary file 3 [file Data_Sheet_1.DOCX]

**Figure S1 The composition differences of gut microbiota between ASD and HC across other taxonomic levels,** related to Figure 3. Bar plots showed the composition differences of gut microbiota at the class (A), order (B) and family (C) levels in two groups. Boxplots revealed the relative abundance of several differential gut microbiota at the class (D), order (E) and family (F) levels between ASD and HC. * p < 0.05, ** p < 0.01; *** p < 0.001.

**Figure S2 Heatmap showed the top 10 up-regulated and 10 down-regulated metabolites of different kinds in ASD compared to HC,** related to Figure 4. (A) Differential metabolites of organic acids. (B) Differential metabolites of amino acids.
